# Depression, Anxiety, and Quality of Life in a Cardiac Rehabilitation Program Without Dedicated Mental Health Resources Post-Myocardial Infarction

**DOI:** 10.3390/jcdd12030092

**Published:** 2025-03-04

**Authors:** Carlos Bertolín-Boronat, Víctor Marcos-Garcés, Héctor Merenciano-González, María Luz Martínez Mas, Josefa Inés Climent Alberola, Nerea Perez, Laura López-Bueno, María Concepción Esteban Argente, María Valls Reig, Ana Arizón Benito, Alfonso Payá Rubio, César Ríos-Navarro, Elena de Dios, Jose Gavara, Manuel F. Jiménez-Navarro, Francisco Javier Chorro, Juan Sanchis, Vicente Bodi

**Affiliations:** 1 Department of Cardiology, Hospital Clinico Universitario de Valencia, 46010 Valencia, Spain; carlosbertolin7@gmail.com (C.B.-B.); hectormeren@gmail.com (H.M.-G.); mluzmmas@comv.es (M.L.M.M.); mvallsr@gmail.com (M.V.R.); francisco.j.chorro@uv.es (F.J.C.); sanchis_juafor@gva.es (J.S.); vicente.bodi@uv.es (V.B.); 2 INCLIVA Health Research Institute, 46010 Valencia, Spain; neere_8@hotmail.com (N.P.); cesar_rios1@hotmail.com (C.R.-N.); 3 Department of Rehabilitation, Hospital Clinico Universitario de Valencia, 46010 Valencia, Spain; inescliment093@gmail.com (J.I.C.A.); laura.lopez@uv.es (L.L.-B.); lulilloluli@yahoo.es (M.C.E.A.); paya_alf@gva.es (A.P.R.); 4 Occupational Risk Prevention Service, Hospital Clinico Universitario de Valencia, 46010 Valencia, Spain; arizon_anaben@gva.es; 5 Network Biomedical Research Center for Cardiovascular Diseases (CIBER-CV), 28029 Madrid, Spain; elenaddll@gmail.com (E.d.D.); mjimeneznavarro@uma.es (M.F.J.-N.); 6 Centre for Biomaterials and Tissue Engineering, Universitat Politènica de València, 46022 Valencia, Spain; jose_4_6_90@hotmail.com; 7 Servicio de Cardiología y Cirugía Cardiovascular-Área del Corazón, Hospital Universitario Virgen de la Victoria, 29010 Málaga, Spain; 8 Instituto de Investigación Biomédica de Málaga y Plataforma en Nanomedicina (IBIMA Plataforma BIONAND), 29590 Málaga, Spain; 9 Departamento de Medicina y Dermatología, Facultad de Medicina, Universidad de Málaga, 29010 Málaga, Spain; 10 Department of Medicine, Faculty of Medicine and Odontology, University of Valencia, 46010 Valencia, Spain

**Keywords:** mental health, myocardial infarction, cardiac rehabilitation, anxiety, depression, quality of life

## Abstract

Anxiety and depression are common after a myocardial infarction (MI), so psychological and psychiatric mental health (MH) interventions are recommended during Cardiac Rehabilitation Programs (CRP). We aim to evaluate anxiety and depression symptoms and quality of life in MI sufferers followed in a CRP without dedicated MH resources. We prospectively included 164 MI patients in our CRP without dedicated MH resources. Patient Health Questionnaire 2-item (PHQ-2) and Generalized Anxiety Disorder 2-item (GAD-2) questionnaires for depression and anxiety screening (altered if ≥3 points) and the 36-Item Short Form Survey Instrument (SF-36) to analyze four MH components and Mental Component Summary (MCS) were assessed at the beginning and after CRP. The mean age was 61.35 ± 10.76 years, and most patients were male (86.6%). A significant improvement in SF-36 mental components (from +5.94 ± 27.98 to +8.31 ± 25 points, *p* < 0.001) and SF-36-MCS (+1.85 ± 10.23 points, *p* = 0.02) was noted, as well as a reduction in depression and anxiety symptoms in PHQ-2 and GAD-2 (*p* < 0.001). However, 33 (20.1%) patients showed a positive screening for depression and/or anxiety at the end of the program. These patients were younger (56.6 ± 8.05 vs. 62.55 ± 11.05 years, *p* = 0.004) and showed significantly worse initial scores of SF-36 mental components, PHQ-2, and GAD-2 (*p* < 0.001). We conclude that a Phase 2 CRP without dedicated MH resources can achieve significant improvements in MH well-being after MI. However, one-fifth of the population had substantial depression and/or anxiety symptoms at the end of the program. This subset, characterized by worse initial MH scores, may benefit from specific MH interventions during CRP.

## 1. Introduction

As the leading cause of mortality worldwide and a substantial burden of morbidity and socio-sanitary costs, cardiovascular disease is a major public health issue [1]. Not only does it cause an increasing number of deaths and disabilities, but it also affects the ability to carry out daily activities and significantly reduces patients’ quality of life (QoL) [2].

Among these conditions, myocardial infarction (MI) stands out as a critical event with significant effects on physical and mental health (MH). In this context, psychological disorders such as anxiety and depression are highly prevalent, markedly impacting patients’ QoL and, in some cases, negatively influencing their clinical progress and prognosis [3,4,5,6].

To perform comprehensive clinical management in the post-MI period, Cardiac Rehabilitation Programs (CRP) are advocated as the best possible intervention to improve patients’ objective and perceived health status and to reduce the risk of subsequent cardiovascular events [7,8,9,10,11]. CRPs provide an individualized exercise program, which can be safely performed in a hospital-centered or home-based program [12]; promote smoking cessation and healthy lifestyle habits; optimize cardiovascular risk factor control; allow for enhanced preventive pharmacotherapy; and effectively improve QoL and prognosis [13]. This multidisciplinary intervention has also been proven to ameliorate anxiety and depression symptoms [4].

Comprehensive psychosocial assessment and subsequent treatment by qualified behavioral health specialists, if required [14], is also considered a core component of the intervention [15], and all MI patients included in a CRP should undergo it. However, even though most CRPs routinely assess MH through depression and anxiety screening tools and QoL scales, they usually lack the resources to provide psychological and psychiatric MH interventions [16,17,18]. In clinical scenarios with limited MH resources, the identification of patients who can benefit most from MH interventions could be useful for prioritizing the allocation of MH resources [19].

Moreover, given the high variability in the services that each CRP offers, and specifically regarding MH resources, it is unclear to what extent MH and QoL improvements are associated with dedicated MH interventions during CRP, with all other non-MH interventions inherent to the CRP, or both.

In our study, we aim to analyze anxiety and depression symptoms and QoL outcomes in MI sufferers followed in a CRP without dedicated MH resources and explore characteristics associated with persistent MH alterations after Phase 2 CRP, which may identify patients who could benefit most from MH interventions.

## 2. Materials and Methods

### 2.1. Population

This study is derived from a prospective registry of ST-segment elevation myocardial infarction (STEMI) or occlusion myocardial infarction (OMI) patients referred to our CRP after discharge from January 2022 to September 2024. Local protocol mandates that all STEMI and OMI patients must be referred to CRP unless contraindicated. Our CRP is centralized in the Hospital Clinico Universitario de Valencia, a high-complexity hospital funded by the Spanish National Health System that provides public universal healthcare to its reference population.

**Figure 1:**
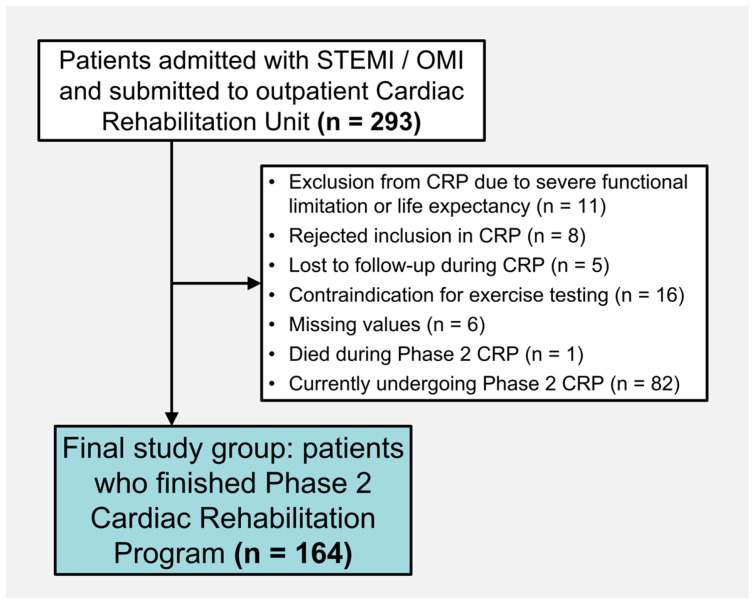
Flowchart of patients included in the study. Abbreviations: CRP = Cardiac Rehabilitation Program. OMI = Occlusion Myocardial Infarction. STEMI = ST-Segment Elevation Acute Myocardial Infarction.

Out of 293 screened candidates and after several reasons for exclusion (Figure 1), the final cohort comprised 164 patients. We registered baseline clinical characteristics, including sex, cardiovascular risk factors, infarct location, Killip class during admission, Global Registry of Acute Coronary Events (GRACE) risk score, and echocardiographic left ventricular ejection fraction (LVEF) before discharge.

### 2.2. Cardiac Rehabilitation Program

Patients included in our CRP undergo a Phase 2 program conducted by a multidisciplinary team of cardiologists, physical medicine and rehabilitation physicians, trained nurses, and physiotherapists. After initial stabilization, conventional or cardiopulmonary exercise testing (C/CPET) is performed, individualized ambulatory training recommendations are provided [12], and additionally, high-risk patients complete 8 to 20 sessions of in-hospital supervised training. Lifestyle education and patient empowerment are promoted during Phase 2. Pharmacological therapy is modified according to patient needs to achieve optimal control of cardiovascular risk factors. After a median follow-up of 7.03 [5.53–8.93] months, C/CPET is repeated, and a holistic assessment of CRP outcomes is performed at the end of Phase 2.

### 2.3. MH Assessment During CRP

All patients included in our CRP undergo MH assessment at the beginning and end of Phase 2. The Patient Health Questionnaire 2-item (PHQ-2) [20] and the Generalized Anxiety Disorder 2-item (GAD-2) [21] questionnaires are used as self-administered screening tools for depression and anxiety symptoms. The PHQ-2 consists of the first two items of the PHQ-9, which are considered the two core criteria for depressive disorders. These items are: (1) Feeling down, depressed, or hopeless, and (2) Little interest or pleasure in doing things. Total scores range from 0 to 6, and cut-off scores of ≥3 are indicative of depression on the PHQ-2 [22]. The GAD-2 consists of the first two items of the GAD-7, which are considered core criteria for diagnosing an anxiety disorder. These items are: (1) Feeling nervous, anxious, or on edge, and (2) Not being able to stop or control worrying. Total scores range from 0 to 6, with a total score ≥3 indicative of a clinically relevant anxiety disorder [22].

We also used the self-administered 36-Item Short Form Survey Instrument (SF-36) as a tool for the health-related quality-of-life assessment [23]. The four MH components of the SF-36 were analyzed separately: emotional role functioning, energy/fatigue, emotional well-being, and social functioning.

Additionally, the Mental Component Summary (MCS) was calculated using a previously described method [24]. A z-score was defined for each MH component of the SF-36 by subtracting the scale mean of a representative sample of the Spanish population [25] from each individual’s scale score and then dividing it by the standard deviation of this reference population. Z-scores were then multiplied by the corresponding factor-scoring coefficient for the MCS scale, and the products of the z-scores and factor-scoring coefficients were then summed together. Subsequently, MCS is linearly transformed into the T-score metric by multiplying the resulting sum by 10 and adding 10 points. An MCS score of <40 points was selected as the cutoff to indicate altered global MH according to the 25th percentile of international SF-36 reference values in patients with ischemic heart disease [26].

### 2.4. MH Interventions During CRP

General psychological counseling is provided to all patients by the multidisciplinary team members at each visit, and relaxation techniques are taught during the initial visit by CRP nurses. Additionally, patients participate in online education by accessing the Open Classroom for Cardiac Rehabilitation (Aula-RC), a teaching program aimed at cardiac rehabilitation patients and developed by the Spanish Society of Cardiology [27]. Aula-RC includes a psychological module focused on emotions, stress management, and relaxation techniques.

Dedicated psychological or psychiatric resources are not available in our CRP, nor are there direct referral pathways to these professionals. In cases where significant anxiety and depressive symptoms are identified, patients are encouraged to request a follow-up appointment with their primary care doctor, who may prescribe anxiolytic or antidepressant medication and/or refer the patient to the local public MH Center, according to their criteria. We registered psychological and psychiatric follow-up and the rate of prescription of anxiolytic and antidepressant medication before and during CRP.

### 2.5. MH Outcomes After CRP

We compared the changes in MH outcomes before and after Phase 2 CRP, i.e., changes in PHQ-2, GAD-2, MH components of SF-36, and SF-36-MCS. We analyzed the proportion of patients with positive screening for depression (PHQ-2 ≥3 points) and anxiety (GAD-2 ≥3 points) and altered SF-36-MCS (<40 points) before and after Phase 2 CRP. We analyzed the predictors of positive screening for depression and/or anxiety at the end of Phase 2 CRP.

### 2.6. Ethics

The study protocol was approved by the Ethics Committee for Drug Research (CEIm) of Hospital Clinico Universitario de Valencia (protocol code: 2019/262 and date of approval: 26 May 2020). This study conforms to the ethical guidelines of the World Medical Association Declaration of Helsinki, and written informed consent was obtained from all subjects.

### 2.7. Statistical Analysis

Normal data distribution was tested with the one-sample Kolmogorov–Smirnov test. Continuous parametric variables were expressed as mean ± standard deviation and were analyzed with Student’s *t*-test for independent or paired samples. Continuous non-parametric variables are shown as the median plus interquartile range and compared using the Mann–Whitney U test for independent samples or the Wilcoxon signed-rank test for paired samples. Qualitative variables are presented as percentages and compared using the chi-square test, Fisher’s exact test, or *z*-test for paired samples.

Statistical significance was considered for 2-tailed *p*-values < 0.05. The SPSS statistical package version 26.0 (IBM Corp., Armonk, NY, USA) was used.

## 3. Results

### 3.1. Cohort Description

The final cohort comprised 164 patients. Baseline characteristics are depicted in Table 1. The mean age was 61.35 ± 10.76 years, most patients were male (86.6%), and hypercholesterolemia was the most prevalent cardiovascular risk factor (89.6%). STEMI was the most frequent presentation (95.7%), the mean LVEF during admission was 52.43 ± 10.55%, and 56 (34.1%) patients had LVEF <50%. Prevalence of anxiety and depression before MI was 13.4% (*n* = 22) and 7.9% (*n* = 13), respectively.

Regarding Phase 2 CRP, a similar distribution across risk categories was noted (low risk, 36.6%; intermediate risk, 28.7%; and high risk, 34.8%), and most patients performed ambulatory training (80.5%), compared to 19.5% who performed additional sessions of supervised in-hospital training. Smoking cessation was achieved in most patients at the end of Phase 2 CRP (from 83, 50.6% to 9, 5.5% active smokers), as well as target LDL-C <55 mg/dL (*n* = 136, 82.9%). High adherence to the Mediterranean diet (*n* = 143, 87.2%) and pharmacological therapy (*n* = 151, 92.1%) was registered after Phase 2 CRP.

### 3.2. MH Outcomes Before and After Phase 2 CRP

During Phase 2 CRP, only a minority of the cohort received psychotherapy (*n* = 8, 4.9%) and psychiatric follow-up (*n* = 9, 5.5%). However, anxiolytic and antidepressant therapy was prescribed to 31 (18.9%) and 28 (17.1%) patients, respectively.

**Figure 2:**
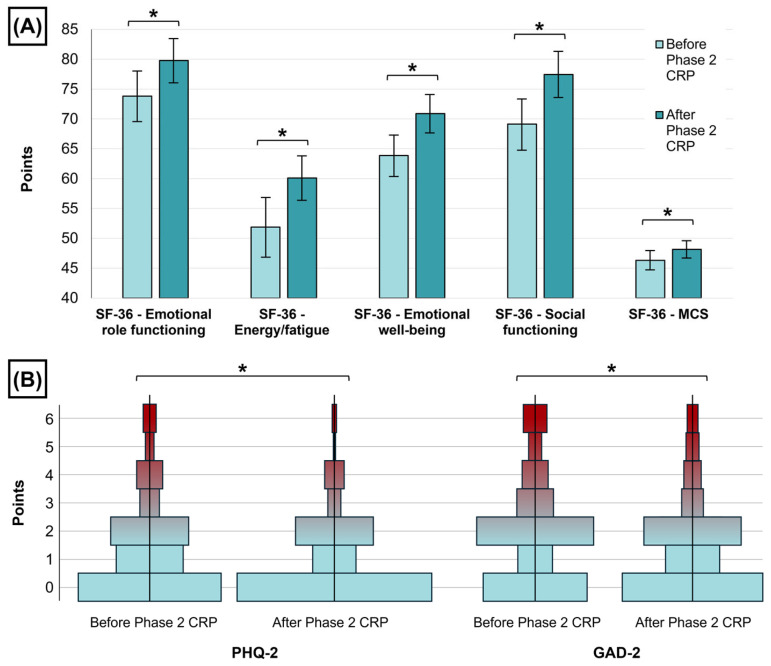
Changes in SF-36 MH components, PHQ-2, and GAD-2 after Phase 2 CRP. (**A**): SF-36 MH-related components and MCS before and after Phase 2 CRP. A significant improvement in all scores was noted (* indicates *p* < 0.05). (**B**): Density plots depicting values in PHQ-2 and GAD-2 scores for depression and anxiety before and after Phase 2 CRP. A significant reduction in depression and anxiety symptoms was noted (* indicates *p* < 0.05). Abbreviations: CRP = Cardiac Rehabilitation Program. GAD-2 = Generalized Anxiety Disorder 2-item. MCS = Mental Component Summary. MH = mental health. PHQ-2 = Patient Health Questionnaire 2-item. SF-36 = 36-Item Short Form Survey Instrument.

After Phase 2 CRP, a significant improvement in SF-36 mental components was noted in the cohort (Table 1, Figure 2A): +5.94 ± 27.98 points in emotional role functioning (*p* = 0.07), +8.24 ± 29.57 in energy/fatigue (*p* < 0.001), +7.01 ± 22.31 points in emotional well-being (*p* < 0.001), and +8.31 ± 25 points in social functioning (*p* < 0.001). Concordantly, SF-36-MCS significantly increased after Phase 2 CRP (+1.85 ± 10.23 points, *p* = 0.02). Also, scores in PHQ-2 and GAD-2 significantly diminished from median 1 [0, 2] to 0 [0, 2] points and median 2 [0, 3] to 1 [0, 2] points (both *p* < 0.001), respectively, indicating a reduction in depressive and anxiety symptoms (Table 1, Figure 2B).

**Figure 3:**
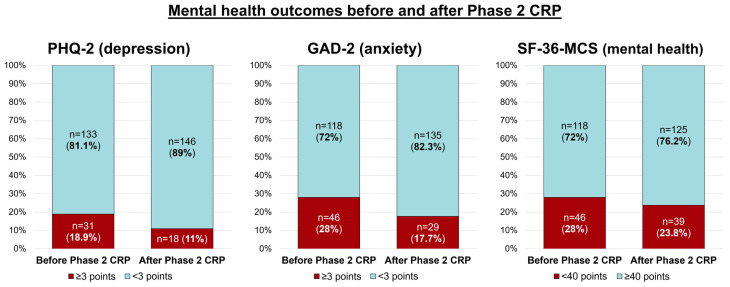
MH outcomes before and after Phase 2 CRP. A reduction in the proportion of patients with positive screening for depression and anxiety (PHQ-2 and GAD-2 ≥3 points) was noted at the end of Phase 2 CRP (*p* = 0.02 and *p* = 0.005, respectively). Also, a trend towards a reduced proportion of patients with altered SF-36-MCS (<40 points) was also noted (*p* = 0.28). Abbreviations: CRP = Cardiac Rehabilitation Program. GAD-2 = Generalized Anxiety Disorder 2-item. MCS = Mental Component Summary. MH = mental health. PHQ-2 = Patient Health Questionnaire 2-item. SF-36 = 36-Item Short Form Survey Instrument.

MH outcomes were analyzed before and after Phase 2 CRP (Figure 3). Positive screening for depression (PHQ-2 ≥3 points) was registered in 31 (18.9%) patients and 18 (11%) patients before and after Phase 2 CRP (*p* = 0.02). The proportion of patients with positive screening for anxiety also diminished at the end of Phase 2 CRP (GAD-2 ≥3 points; *n* = 46, 28% vs. *n* = 29, 17.7%; *p* = 0.005). A trend towards a reduced proportion of patients with altered SF-36-MCS (<40 points) was also noted at the end of Phase 2 CRP, although not statistically significant (*n* = 46, 28% vs. *n* = 39, 23.8%; *p* = 0.28).

### 3.3. Depression and Anxiety Symptoms After Phase 2 CRP

Despite the intervention during Phase 2 CRP, a non-neglectable proportion of patients (*n* = 33, 20.1%) showed a positive screening for depression (*n* = 4, 2.4%), anxiety (*n* = 15, 10.3%), or both (*n* = 14, 8.5%) at the end of the program (Table 1).

This subset of patients was younger (56.6 ± 8.05 vs. 62.55 ± 11.05 years, *p* = 0.004), and a trend towards a higher proportion of supervised in-hospital training was noted (30.3% vs. 16.8%, *p* = 0.08). However, a trend towards a lower increase in weekly physical activity was registered in this population (+1374 [406–2986.5] vs. +2148 [513–4599] METS/week, p = 0.09), as well as less weekly exercising at the end of CRP (2772 [1713–5359.5] METS/week vs. 4005 [2331–7074] METS/week, *p* = 0.02). Also, patients with positive depression and/or anxiety screening at the end of Phase 2 showed a trend towards a lower likelihood of achieving target LDL-C <55 mg/dL (*n* = 24, 72.7% vs. *n* = 112, 85.5%, *p* = 0.08).

Previous history of depression and anxiety before MI was higher in this population (18.2% vs. 5.3% and 24.2% vs. 10.7%, respectively). These patients showed significantly lower initial scores in SF-36 mental components and SF-36-MCS (*p* < 0.001 for all comparisons), which remained virtually unchanged at the end of Phase 2 CRP. Initial PHQ-2 (median 2 [1, 4] points) and GAD-2 (median 3 [2, 4] points) scores were high, indicating significant depression and anxiety symptoms, and these scores didn’t change at the end of Phase 2 CRP (median 3 [2, 5] and 4 [3, 5] points, respectively).

## 4. Discussion

In our study of post-MI patients who underwent Phase 2 CRP, we demonstrate that after the acute event, nearly two out of ten and three out of ten patients have a positive screening for depression and anxiety, respectively. Our findings show that Phase 2 CRP without dedicated MH resources has a significant and positive impact on MH improvement within the cohort. However, two out of ten patients continue to exhibit substantial depressive and/or anxiety symptoms at the end of the intervention. This specific subset is characterized by significantly worse initial depression, anxiety, and MH-related QoL scores, potentially helping to identify those individuals who may benefit most from dedicated MH interventions during CRP.

### 4.1. Mental Health After MI

Depression and anxiety are common among patients who experience an MI [28,29]. Studies have reported a 20% to 40% prevalence of depression and anxiety in MI sufferers [30,31,32], and these conditions can also emerge as newly diagnosed following the event, with a 5- to 7-fold increased risk of developing anxiety and depression described after MI [33]. Moreover, a bidirectional relationship between stress or anxiety and depression has been noted in these patients [28].

Not only can depression and anxiety significantly affect QoL after MI [34,35], but they have also been consistently associated with lower adherence to guideline-directed pharmacological therapy and other secondary prevention measures [28,36,37,38], as well as a higher risk of recurrent cardiac events [5,39,40,41,42,43] and worse physical performance [6]. Thus, systematic screening for anxiety and depression is recommended after an MI due to their high prevalence and clinical implications [14,44].

### 4.2. Cardiac Rehabilitation and Mental Health

CRPs provide the best possible framework for holistic management after an MI [7,8,9,10,11]. Among other clinical interventions such as cardiological therapy optimization, nurse-led education in healthy lifestyle habits, and individualized exercise training, they also allow for MH status evaluation, anxiety and depression screening, and tailored MH interventions when available [14].

The identification of patients with significant anxiety and/or depression symptoms is a necessary first step toward implementing MH interventions. Following current recommendations, most CRPs routinely assess MH using depression and anxiety screening tools and QoL scales [16,17,18]. In our study, we found that routine screening at the first assessment during Phase 2 CRP indicates that nearly 20% and 30% of the cohort potentially may present with significant depression and anxiety symptoms based on PHQ-2 and GAD-2 scores, respectively. A similar prevalence has previously been reported in the literature [30,31,32], and this rate nearly doubles the prevalence of a previous clinical diagnosis of depression and/or anxiety in our cohort.

### 4.3. MH Interventions During CRPs

Considering several types of cardiological conditions, CRPs have shown an improvement in anxiety and depression symptoms and MH-related QoL scores [4,7,45]. This amelioration in MH status has been indistinctly noted in both center-based and home-based or ambulatory CRPs [46,47] and has generally been analyzed in aggregated populations of post-MI patients, irrespective of whether they exhibited significant depression and/or anxiety symptoms or not.

MH resources during CRPs can either be non-specifically offered to all patients undergoing CRPs or directed towards the most affected individuals. Despite many centers lacking the availability of these MH interventions [16,17], evidence shows that their incorporation into CRPs can translate into significant clinical benefits [48,49]. Stress management training through group interventions, including relaxation techniques, cognitive restructuring, communication and assertiveness skills, and problem-solving, has shown significant improvements in MH, QoL, and medium-term prognosis [50]. Other interventions focused on group metacognitive therapy [51] and even structured patient education programs [52] have also demonstrated MH improvements. Psychopharmacological therapy also improves mental well-being, when indicated [53].

No specific MH interventions were performed as a component of our Phase 2 CRP, and psychotherapy and psychiatric follow-up outside the CRP were uncommon, although the prescription of anxiolytic and antidepressant pharmacotherapy by primary care physicians increased post-MI. Despite the lack of MH interventions inside the CRP, we noted a significant improvement in depression and anxiety symptoms and MH-related QoL scores.

Apart from the beneficial effects of therapy by qualified behavioral health specialists [14] and psychopharmacology provided outside CRPs, several other factors can contribute to MH amelioration during CRP. Exercise training, which is systematically prescribed during Phase 2 CRP, is known to have direct effects on overall well-being and self-confidence, alleviating depressive and anxiety symptoms [54]. Indeed, we noted that patients who exercised more at the end of CRP and those with larger increments in weekly physical activity according to the IPAQ questionnaire less frequently showed a positive depression/anxiety screening after Phase 2 CRP.

Additionally, CRPs enhance self-efficacy, which can improve a patient’s ability to control symptoms, exercise regularly, adhere to medication regimens, and adopt a healthy lifestyle, thus improving psychological well-being [19]. Apart from that, clinical care provided by the multidisciplinary team of non-MH professionals during CRP in the first months after MI can also have a beneficial psychotherapeutic effect, although it can often remain overlooked.

### 4.4. Prioritization of MH Resources During CRP

Even though our study provides evidence regarding MH improvements in the post-MI period even when no specific MH resources are available during Phase 2 CRP, in scenarios where these resources are available but scarce, it would be preferable to redirect them to patients who are most likely to obtain a significant clinical benefit from them.

To try to identify this subset, in our cohort, we studied which patients remained significantly symptomatic despite our CRP intervention, i.e., those showing a positive screening for anxiety and/or depression at the end of Phase 2. This subgroup, which accounted for more than 20% of the cohort, displayed a younger age, a more prevalent history of depression and anxiety before MI, and, most importantly, worse scores on anxiety and depression screening assessed by GAD-2 and PHQ-2 questionnaires and MH-related QoL components of SF-36.

It would undoubtedly be beneficial to incorporate psychosocial evaluations during CRP to assess all factors (e.g., socioeconomic status, personality traits, social support, illness beliefs, coping mechanisms, etc.) that can worsen MH after MI [14,35]. Several other factors, such as low socioeconomic status, social isolation, and lack of support, increase the risk of anxiety and depression after a cardiac event [55]. However, alongside these generally complex evaluations, screening tools for anxiety and depression and QoL scores could be a practical and straightforward method to identify patients who are likely to remain symptomatic after CRP and who may benefit from specific MH interventions.

Due to the rising prevalence of cardiovascular disease and in the current era of increasing sociosanitary costs [1], the incorporation of this strategy into CRPs could help rationalize MH resources and increase the overall availability of CRPs for many sufferers of cardiovascular conditions. Nonetheless, prospective studies should confirm our data and delve into the usefulness of such a screening method.

### 4.5. Limitations

Our study has several limitations that should be acknowledged. First, it is based on an observational, prospective cohort, making it susceptible to selection bias. Moreover, only STEMI and OMI patients were included in the registry, which limits its representativeness of the whole MI spectrum. The lack of a control group due to ethical reasons prevented us from drawing definitive conclusions regarding the effect of a Phase 2 CRP in MH improvement after MI. Although our results are consistent with previous studies [4,7,45,46], a fair degree of natural recovery exists in this population after an early adjustment phase [56], which may not be related to CRP. Additionally, we used MH questionnaires, which are inherently subjective, evaluate only a limited number of MH parameters, and cannot substitute for a formal diagnosis of depression and/or anxiety by qualified MH professionals. Patient follow-up was conducted only until the end of Phase 2 CRP, so long-term changes in MH status were not analyzed. Finally, the cohort was single-center, so external validation is needed.

## 5. Conclusions

In our prospective cohort of post-MI individuals, we demonstrate that a Phase 2 CRP without dedicated MH interventions can achieve significant improvements in MH well-being after the acute event. However, one-fifth of the population had substantial depression and/or anxiety symptoms at the end of the program. This specific subset, characterized by worse initial MH scores, may identify individuals who could benefit from specific MH interventions during CRP.

## Figures and Tables

**Table 1:** Baseline characteristics of the cohort and in patients with and without positive depression/anxiety screening after Phase 2 CRP.

	All Patients (*n* = 164)	Negative Depression/Anxiety Screening After Phase 2 CRP (*n* = 131)	Positive Depression/Anxiety Screening After Phase 2 CRP (*n* = 33)	*p*-Value
**Clinical variables**				
Age (years)	61.35 ± 10.76	62.55 ± 11.05	56.6 ± 8.05	0.004
Male sex (%)	142 (86.6)	115 (87.8)	27 (81.8)	0.37
Hypercholesterolemia (%)	147 (89.6)	116 (88.5)	31 (93.9)	0.36
Hypertension (%)	90 (54.9)	73 (55.7)	17 (51.5)	0.66
Diabetes mellitus (%)	33 (20.1)	29 (22.1)	4 (12.1)	0.2
Killip class ≥2 (%)	45 (27.4)	38 (29)	7 (21.2)	0.37
GRACE risk score	115.85 ± 29.25	116.68 ± 29.8	112.58 ± 27.11	0.47
Infarct location				0.33
Anterior (%)	72 (43.9)	59 (45)	13 (39.4)	
Inferior (%)	73 (44.5)	57 (43.5)	16 (48.5)	
Lateral (%)	12 (7.3)	8 (6.1)	4 (12.1)	
OMI (%)	7 (4.3)	7 (5.3)	0 (0)	
LVEF (%)	52.43 ± 10.55	52.5 ± 10.6	52.12 ± 10.5	0.85
LVEF <50% (%)	56 (34.1)	45 (34.4)	11 (33.3)	0.91
Risk stratification for CRP (%)				0.47
Low risk	60 (36.6)	48 (36.6)	12 (36.4)	
Intermediate risk	47 (28.7)	40 (30.5)	7 (21.2)	
High risk	57 (34.8)	43 (32.8)	14 (42.4)	
Exercise training modality during CRP				0.08
Ambulatory training (%)	132 (80.5)	109 (83.2)	23 (69.7)	
Supervised in-hospital training (%)	32 (19.5)	22 (16.8)	10 (30.3)	
**Cardiovascular risk factors**				
Smoking habit before CRP (%)	83 (50.6)	65 (49.6)	18 (54.5)	0.61
Smoking habit after CRP (%)	9 (5.5)	7 (5.3)	2 (6.1)	0.87
Systolic pressure (mmHg)				
Before CRP	125.05 ± 16.08	125.53 ± 16.19	123.15 ± 15.71	0.45
After CRP	114.96 ± 10.08	115.29 ± 10.03	113.64 ± 10.33	0.4
Mean change	−10.09 ± 14 *	−10.24 ± 13.35 *	−9.52 ± 16.54 *	0.79
LDL-C (mg/dL)				
Before CRP	101.85 ± 13.95	99.24 ± 34.31	111.88 ± 42.66	0.08
After CRP	44.43 ± 13.95	43.84 ± 13.37	46.76 ± 16.05	0.28
Mean change	−57.29 ± 36.43 *	−55.26 ± 34.67 *	−65.12 ± 42.22 *	0.17
LDL-C <55 mg/dL after CRP (%)	136 (82.9)	112 (85.5)	24 (72.7)	0.08
Weight (kg)				
Before CRP	80.7 ± 15.18	80.74 ± 15.69	80.55 ± 13.21	0.95
After CRP	79.15 ± 14.55	79.23 ± 14.93	78.8 ± 13.18	0.88
Mean change	−1.56 ± 5.99 *	−1.51 ± 5.58 *	−1.75 ± 7.49	0.84
BMI				
Before CRP	27.72 ± 4.62	27.67 ± 4.6	27.97 ± 4.76	0.73
After CRP	27.21 ± 4.45	27.15 ± 4.4	27.42 ± 4.72	0.76
Mean change	−0.52 ± 2.02 *	−0.51 ± 1.88 *	−0.55 ± 2.53	0.92
BMI ≥30 before CRP (%)	39 (23.8)	30 (22.9)	9 (27.3)	0.6
BMI ≥30 after CRP (%)	31 (18.9)	26 (19.8)	5 (15.2)	0.54
PREDIMED ≥8 points before CRP (%)	118 (72)	93 (71)	25 (75.8)	0.59
PREDIMED ≥8 points after CRP (%)	143 (87.2)	113 (86.3)	30 (90.9)	0.48
Therapeutic adherence before CRP (4 points in Morisky-Green, %)	130 (79.3)	104 (79.4)	26 (78.8)	0.94
Therapeutic adherence after CRP (4 points in Morisky-Green, %)	151 (92.1)	120 (91.6)	31 (93.9)	0.66
**MH and QoL outcomes**				
Previous history of anxiety	22 (13.4)	14 (10.7)	8 (24.2)	0.04
Previous history of depression	13 (7.9)	7 (5.3)	96 (18.2)	0.02
SF-36—Emotional role functioning (points)				
Before CRP	73.83 ± 27.98	78.37 ± 25.59	55.81 ± 30.16	<0.001
After CRP	79.78 ± 24.23	86.39 ± 18.67	53.54 ± 26.19	<0.001
Mean change	5.94 ± 27.98 *	8.01 ± 27.67 *	−2.27 ± 28.13	0.06
SF-36—Energy/fatigue (points)				
Before CRP	51.88 ± 32.98	56.69 ± 33.72	32.77 ± 21.2	<0.001
After CRP	60.12 ± 24.42	66.48 ± 20.02	34.86 ± 24.22	<0.001
Mean change	8.24 ± 29.57 *	9.79 ± 31.62 *	2.09 ± 18.54	0.18
SF-36—Emotional well-being (points)				
Before CRP	63.87 ± 23.03	68.17 ± 21.68	46.82 ± 20.42	<0.001
After CRP	70.88 ± 21.15	77.71 ± 16.26	43.79 ± 16.01	<0.001
Mean change	7.01 ± 22.31 *	9.54 ± 22.39 *	−3.03 ± 19.2	0.004
SF-36—Social functioning (points)				
Before CRP	69.13 ± 28.19	73.38 ± 26.47	52.27 ± 28.89	<0.001
After CRP	77.44 ± 25.34	84.06 ± 20.04	51.14 ± 27.31	<0.001
Mean change	8.31 ± 25 *	10.69 ± 27.87 *	−1.14 ± 31.15	0.04
SF-36—Mental component summary (MCS)				
Before CRP	46.31 ± 10.64	48.21 ± 10.38	38.77 ± 8.1	<0.001
After CRP	48.16 ± 9.51	50.94 ± 7.62	37.1 ± 8.18	<0.001
Mean change	1.85 ± 10.23 *	2.73 ± 10.65 *	−1.67 ± 7.5	0.03
PHQ-2 (points)				
Before CRP	1 [0, 2]	1 [0, 2]	2 [1, 4]	<0.001
After CRP	0 [0, 2]	0 [0, 1]	3 [2, 4]	<0.001
Median change	0 [0, −1] *	0 [0, −1] *	0 [1, −1]	0.03
GAD-2 (points)				
Before CRP	2 [0, 3]	2 [0, 2]	3 [2, 5]	<0.001
After CRP	1 [0, 2]	1 [0, 2]	4 [3, 5]	<0.001
Median change	0 [0, −1] *	0 [0, −2] *	0 [2, −1]	0.005
**Physical fitness variables**				
IPAQ (METS/week)				
Before CRP	1386 [668.25–2748.75]	1386 [693–2772]	1188 [495–2722.5]	0.23
After CRP	3662 [2106–6484.5]	4005 [2331–7074]	2772 [1713–5359.5]	0.02
Median change	1845 [502.5–4370.25] *	2148 [513–4599] *	1374 [406–2986.5] *	0.09
Peak VO2 (ml/kg/min)				
Before CRP	25.93 ± 9.17	25.71 ± 8.95	26.79 ± 10.11	0.55
After CRP	30.05 ± 10.36	30.07 ± 10.46	29.95 ± 10.13	0.95
Mean change	4.12 ± 5.25 *	4.36 ± 5.2 *	3.16 ± 5.42 *	0.24
**Psychological and psychiatric therapy before Phase 2 CRP**				
Psychotherapy	8 (4.9)	5 (3.8)	3 (9.1)	0.21
Psychiatric follow-up	8 (4.9)	6 (4.6)	2 (6.1)	0.72
Anxiolytic pharmacotherapy	19 (11.6)	16 (12.2)	3 (9.1)	0.62
Antidepressant pharmacotherapy	16 (9.8)	11 (8.4)	5 (15.2)	0.24
**Psychological and psychiatric therapy after Phase 2 CRP**				
Psychotherapy	8 (4.9)	3 (2.3)	5 (15.2)	0.002
Psychiatric follow-up	9 (5.5)	4 (3.1)	5 (15.2)	0.006
Anxiolytic pharmacotherapy	31 (18.9)	20 (15.3)	11 (33.3)	0.02
Antidepressant pharmacotherapy	28 (17.1)	15 (11.5)	13 (39.4)	<0.001

* *p* < 0.05 for paired samples comparison. Abbreviations: BMI = body mass index. CRP = Cardiac Rehabilitation Program. GAD-2 = Generalized Anxiety Disorder 2-item. GRACE = Global Registry of Acute Coronary Events. IPAQ = International Physical Activity Questionnaire. LDL-C = low-density lipoprotein cholesterol. LVEF = left ventricular ejection fraction. MCS = Mental Component Summary. METS = metabolic equivalents. MH = mental health. PHQ-2 = Patient Health Questionnaire 2-item. QoL = quality of life. SF-36 = 36-Item Short Form Survey Instrument. VO2 = oxygen consumption.

## Data Availability

The data presented in this study are available on request from the corresponding authors. The data are not publicly available due to ethical restrictions.

## Abbreviations

The following abbreviations are used in this manuscript:

C/CPET	Conventional or Cardiopulmonary Exercise Testing
CRP	Cardiac Rehabilitation Program
GAD-2	Generalized Anxiety Disorder 2-item
GRACE	Global Registry of Acute Coronary Events
IPAQ	International Physical Activity Questionnaire
LDL-C	Low-Density Lipoprotein Cholesterol
LVEF	Left Ventricular Ejection Fraction
MCS	Mental Component Summary
MH	Mental Health
MI	Myocardial Infarction
OMI	Occlusion Myocardial Infarction
PHQ-2	Patient Health Questionnaire 2-item
QoL	Quality of Life
SF-36	36-Item Short Form Survey Instrument
SPSS	Statistical Package for the Social Sciences
STEMI	ST-segment Elevation Myocardial Infarction

## References

[1] Martin S.S., Aday A.W., Almarzooq Z.I., Anderson C.A.M., Arora P., Avery C.L., Baker-Smith C.M., Barone Gibbs B., Beaton A.Z., Boehme A.K. (2024). 2024 Heart Disease and Stroke Statistics: A Report of US and Global Data From the American Heart Association. Circulation.

[2] Lui J.N.M., Williams C., Keng M.J., Hopewell J.C., Sammons E., Chen F., Gray A., Bowman L., Landray S.M.J., Mihaylova B. (2023). Impact of New Cardiovascular Events on Quality of Life and Hospital Costs in People with Cardiovascular Disease in the United Kingdom and United States. J. Am. Heart Assoc..

[3] Watkins L.L., Koch G.G., Sherwood A., Blumenthal J.A., Davidson J.R.T., O’Connor C., Sketch M.H. (2013). Association of Anxiety and Depression With All-Cause Mortality in Individuals With Coronary Heart Disease. J. Am. Heart Assoc..

[4] Zheng X., Zheng Y., Ma J., Zhang M., Zhang Y., Liu X., Chen L., Yang Q., Sun Y., Wu J. (2019). Effect of Exercise-Based Cardiac Rehabilitation on Anxiety and Depression in Patients with Myocardial Infarction: A Systematic Review and Meta-Analysis. Heart Lung.

[5] Meijer A., Conradi H.J., Bos E.H., Thombs B.D., Van Melle J.P., De Jonge P. (2011). Prognostic Association of Depression Following Myocardial Infarction with Mortality and Cardiovascular Events: A Meta-Analysis of 25 Years of Research. Gen. Hosp. Psychiatry.

[6] Sakamoto M., Suematsu Y., Yano Y., Kaino K., Teshima R., Matsuda T., Fujita M., Tazawa R., Fujimi K., Miura S. (2022). Depression and Anxiety Are Associated with Physical Performance in Patients Undergoing Cardiac Rehabilitation: A Retrospective Observational Study. J. Cardiovasc. Dev. Dis..

[7] Dibben G.O., Faulkner J., Oldridge N., Rees K., Thompson D.R., Zwisler A.-D., Taylor R.S. (2023). Exercise-Based Cardiac Rehabilitation for Coronary Heart Disease: A Meta-Analysis. Eur. Heart J..

[8] Byrne R.A., Rossello X., Coughlan J.J., Barbato E., Berry C., Chieffo A., Claeys M.J., Dan G.-A., Dweck M.R., Galbraith M. (2023). 2023 ESC Guidelines for the Management of Acute Coronary Syndromes. Eur. Heart J..

[9] Virani S.S., Newby L.K., Arnold S.V., Bittner V., Brewer L.C., Demeter S.H., Dixon D.L., Fearon W.F., Hess B., Johnson H.M. (2023). 2023 AHA/ACC/ACCP/ASPC/NLA/PCNA Guideline for the Management of Patients With Chronic Coronary Disease: A Report of the American Heart Association/American College of Cardiology Joint Committee on Clinical Practice Guidelines. Circulation.

[10] Vrints C., Andreotti F., Koskinas K.C., Rossello X., Adamo M., Ainslie J., Banning A.P., Budaj A., Buechel R.R., Chiariello G.A. (2024). 2024 ESC Guidelines for the Management of Chronic Coronary Syndromes. Eur. Heart J..

[11] Mansilla-Chacón M., Gómez-Urquiza J.L., Martos-Cabrera M.B., Albendín-García L., Romero-Béjar J.L., Cañadas-De La Fuente G.A., Suleiman-Martos N. (2021). Effects of Supervised Cardiac Rehabilitation Programmes on Quality of Life among Myocardial Infarction Patients: A Systematic Review and Meta-Analysis. J. Cardiovasc. Dev. Dis..

[12] Stefanakis M., Batalik L., Antoniou V., Pepera G. (2022). Safety of Home-Based Cardiac Rehabilitation: A Systematic Review. Heart Lung.

[13] Taylor R.S., Dalal H.M., McDonagh S.T.J. (2021). The Role of Cardiac Rehabilitation in Improving Cardiovascular Outcomes. Nat. Rev. Cardiol..

[14] Hughes J.W., Serber E.R., Kuhn T. (2022). Psychosocial Management in Cardiac Rehabilitation: Current Practices, Recommendations, and Opportunities. Prog. Cardiovasc. Dis..

[15] Brown T.M., Pack Q.R., Aberegg E., Brewer L.C., Ford Y.R., Forman D.E., Gathright E.C., Khadanga S., Ozemek C., Thomas R.J. (2024). Core Components of Cardiac Rehabilitation Programs: 2024 Update: A Scientific Statement From the American Heart Association and the American Association of Cardiovascular and Pulmonary Rehabilitation. Circulation.

[16] Bush M., Evenson K.R., Aylward A., Cyr J.M., Kucharska-Newton A. (2023). Psychosocial Services Provided by Licensed Cardiac Rehabilitation Programs. Front. Rehabil. Sci..

[17] Jackson A.C., Le Grande M.R., Higgins R.O., Rogerson M., Murphy B.M. (2017). Psychosocial Screening and Assessment Practice within Cardiac Rehabilitation: A Survey of Cardiac Rehabilitation Coordinators in Australia. Heart Lung Circ..

[18] Cahill M.C., Bilanovic A., Kelly S., Bacon S., Grace S.L. (2015). Screening for Depression in Cardiac Rehabilitation: A REVIEW. J. Cardiopulm. Rehabil. Prev..

[19] McKenzie K.M., Park L.K., Lenze E.J., Montgomery K., Rashdi S., Deych E., Stranczek N.A., McKenzie E.J., Rich M.W., Garr Barry V. (2022). A Prospective Cohort Study of the Impact of Outpatient Intensive Cardiac Rehabilitation on Depression and Cardiac Self-Efficacy. Am. Heart J. Plus Cardiol. Res. Pract..

[20] Levis B., Sun Y., He C., Wu Y., Krishnan A., Bhandari P.M., Neupane D., Imran M., Brehaut E., Negeri Z. (2020). Accuracy of the PHQ-2 Alone and in Combination With the PHQ-9 for Screening to Detect Major Depression: Systematic Review and Meta-Analysis. JAMA.

[21] Hlynsson J.I., Carlbring P. (2024). Diagnostic Accuracy and Clinical Utility of the PHQ-2 and GAD-2: A Comparison with Long-Format Measures for Depression and Anxiety. Front. Psychol..

[22] Staples L.G., Dear B.F., Gandy M., Fogliati V., Fogliati R., Karin E., Nielssen O., Titov N. (2019). Psychometric Properties and Clinical Utility of Brief Measures of Depression, Anxiety, and General Distress: The PHQ-2, GAD-2, and K-6. Gen. Hosp. Psychiatry.

[23] García-Sánchez E., Santamaría-Peláez M., Benito Figuerola E., Carballo García M.J., Chico Hernando M., García García J.M., González-Bernal J.J., González-Santos J. (2024). Comparison of SF-36 and RAND-36 in Cardiovascular Diseases: A Reliability Study. J. Clin. Med..

[24] Laucis N.C., Hays R.D., Bhattacharyya T. (2015). Scoring the SF-36 in Orthopaedics: A Brief Guide. J. Bone Jt. Surg..

[25] López-García E., Banegas J.R., Pérez-Regadera A.G., Gutiérrez-Fisac J.L., Alonso J., Rodríguez-Artalejo F. (2003). Valores de referencia de la versión española del Cuestionario de Salud SF-36 en población adulta de más de 60 años. Med. Clínica.

[26] Huber A., Oldridge N., Höfer S. (2016). International SF-36 Reference Values in Patients with Ischemic Heart Disease. Qual. Life Res..

[27] Inicio—Pacientes—Aula Abierta RC. https://pacientes.aularc.es/.

[28] Rao A., Zecchin R., Newton P., Phillips J., DiGiacomo M., Denniss A., Hickman L. (2020). The Prevalence and Impact of Depression and Anxiety in Cardiac Rehabilitation: A Longitudinal Cohort Study. Eur. J. Prev. Cardiol..

[29] Sreenivasan J., Khan M.S., Khan S.U., Hooda U., Aronow W.S., Panza J.A., Levine G.N., Commodore-Mensah Y., Blumenthal R.S., Michos E.D. (2021). Mental Health Disorders among Patients with Acute Myocardial Infarction in the United States. Am. J. Prev. Cardiol..

[30] Feng L., Li L., Liu W., Yang J., Wang Q., Shi L., Luo M. (2019). Prevalence of Depression in Myocardial Infarction: A PRISMA-Compliant Meta-Analysis. Medicine.

[31] Roest A.M., Martens E.J., Denollet J., De Jonge P. (2010). Prognostic Association of Anxiety Post Myocardial Infarction With Mortality and New Cardiac Events: A Meta-Analysis. Psychosom. Med..

[32] Lian Y., Xiang J., Wang X., Kaminga A.C., Chen W., Lai Z., Dai W., Yang J. (2022). Prevalence of Moderate to Severe Anxiety Symptoms among Patients with Myocardial Infarction: A Meta-Analysis. Psychiatr. Q..

[33] Feng H.-P., Chien W.-C., Cheng W.-T., Chung C.-H., Cheng S.-M., Tzeng W.-C. (2016). Risk of Anxiety and Depressive Disorders in Patients with Myocardial Infarction: A Nationwide Population-Based Cohort Study. Medicine.

[34] Hosseini S.H., Ghaemian A., Mehdizadeh E., Ashraf H. (2014). Contribution of Depression and Anxiety to Impaired Quality of Life in Survivors of Myocardial Infarction. Int. J. Psychiatry Clin. Pract..

[35] Garrels E., Kainth T., Silva B., Yadav G., Gill G., Salehi M., Gunturu S. (2023). Pathophysiological Mechanisms of Post-Myocardial Infarction Depression: A Narrative Review. Front. Psychiatry.

[36] Lapa M.E., Swabe G.M., Rollman B.L., Muldoon M.F., Thurston R.C., Magnani J.W. (2022). Assessment of Depression and Adherence to Guideline-Directed Medical Therapies Following Percutaneous Coronary Intervention. JAMA Netw. Open.

[37] Ashour A.M., Masa’deh R., Hamaideh S.H., Elshatarat R.A., Yacoub M.I., Almagharbeh W.T., Alhejaili A.A., Alshahrani B.D., Sobeh D.E., Eltayeb M.M. (2024). Examining the Influence of Anxiety and Depression on Medication Adherence among Patients Diagnosed with Acute Myocardial Infarction. BMC Psychol..

[38] Ambrosetti M., Abreu A., Corrà U., Davos C.H., Hansen D., Frederix I., Iliou M.C., Pedretti R.F.E., Schmid J.-P., Vigorito C. (2021). Secondary Prevention through Comprehensive Cardiovascular Rehabilitation: From Knowledge to Implementation. 2020 Update. A Position Paper from the Secondary Prevention and Rehabilitation Section of the European Association of Preventive Cardiology. Eur. J. Prev. Cardiol..

[39] Flygare O., Boberg J., Rück C., Hofmann R., Leosdottir M., Mataix-Cols D., De La Cruz L.F., Richman P., Wallert J. (2023). Association of Anxiety or Depression with Risk of Recurrent Cardiovascular Events and Death after Myocardial Infarction: A Nationwide Registry Study. Int. J. Cardiol..

[40] Leissner P., Held C., Humphries S., Rondung E., Olsson E.M.G. (2024). Association of Anxiety and Recurrent Cardiovascular Events: Investigating Different Aspects of Anxiety. Eur. J. Cardiovasc. Nurs..

[41] Cha S., Chang W.K., Lee K., Han K., Paik N.-J., Kim W.-S. (2024). Prevalence and Impact of Depression and Anxiety among Older Myocardial Infarction Survivors: A Nationwide Cohort Study. J. Affect. Disord..

[42] Kim J.-M., Stewart R., Kim J.-W., Kang H.-J., Kim S.-W., Shin I.-S., Hong Y.J., Ahn Y., Jeong M.H., Yoon J.-S. (2020). Impact of Depression at Early and Late Phases Following Acute Coronary Syndrome on Long-Term Cardiac Outcomes. J. Affect. Disord..

[43] Wen Y., Yang Y., Shen J., Luo S. (2021). Anxiety and Prognosis of Patients with Myocardial Infarction: A Meta-analysis. Clin. Cardiol..

[44] Helmark C., Harrison A., Pedersen S.S., Doherty P. (2022). Systematic Screening for Anxiety and Depression in Cardiac Rehabilitation—Are We There Yet?. Int. J. Cardiol..

[45] Dibben G., Faulkner J., Oldridge N., Rees K., Thompson D.R., Zwisler A.-D., Taylor R.S. (2021). Exercise-Based Cardiac Rehabilitation for Coronary Heart Disease. Cochrane Database Syst. Rev..

[46] Bravo-Escobar R., González-Represas A., Gómez-González A.M., Heredia-Torres Á. (2021). Effectiveness of E-Health Cardiac Rehabilitation Program on Quality of Life Associated with Symptoms of Anxiety and Depression in Moderate-Risk Patients. Sci. Rep..

[47] Anderson L., Sharp G.A., Norton R.J., Dalal H., Dean S.G., Jolly K., Cowie A., Zawada A., Taylor R.S. (2017). Home-Based versus Centre-Based Cardiac Rehabilitation. Cochrane Database Syst. Rev..

[48] Wrzeciono A., Mazurek J., Cieślik B., Kiper P., Gajda R., Szczepańska-Gieracha J. (2024). Psychologically-Enhanced Cardiac Rehabilitation for Psychological and Functional Improvement in Patients with Cardiovascular Disease: A Systematic Review with Meta-Analysis and Future Research Directions. Physiotherapy.

[49] Albus C., Herrmann-Lingen C., Jensen K., Hackbusch M., Münch N., Kuncewicz C., Grilli M., Schwaab B., Rauch B., German Society of Cardiovascular Prevention & Rehabilitation (DGPR) (2019). Additional Effects of Psychological Interventions on Subjective and Objective Outcomes Compared with Exercise-Based Cardiac Rehabilitation Alone in Patients with Cardiovascular Disease: A Systematic Review and Meta-Analysis. Eur. J. Prev. Cardiol..

[50] Blumenthal J.A., Sherwood A., Smith P.J., Watkins L., Mabe S., Kraus W.E., Ingle K., Miller P., Hinderliter A. (2016). Enhancing Cardiac Rehabilitation With Stress Management Training: A Randomized, Clinical Efficacy Trial. Circulation.

[51] Wells A., Reeves D., Capobianco L., Heal C., Davies L., Heagerty A., Doherty P., Fisher P. (2021). Improving the Effectiveness of Psychological Interventions for Depression and Anxiety in Cardiac Rehabilitation: PATHWAY—A Single-Blind, Parallel, Randomized, Controlled Trial of Group Metacognitive Therapy. Circulation.

[52] Zhamaliyeva L.M., Zhamankulova D.G., Abenova N.A., Koshmaganbetova G.K. (2023). Educational Intervention Effects on Depression and Anxiety in Patients after Myocardial Infarction: A Randomized Controlled Trial. J. Cardiovasc. Dev. Dis..

[53] Zambrano J., Celano C.M., Januzzi J.L., Massey C.N., Chung W., Millstein R.A., Huffman J.C. (2020). Psychiatric and Psychological Interventions for Depression in Patients With Heart Disease: A Scoping Review. J. Am. Heart Assoc..

[54] Blumenthal J.A., Rozanski A. (2023). Exercise as a Therapeutic Modality for the Prevention and Treatment of Depression. Prog. Cardiovasc. Dis..

[55] Murphy B., Le Grande M., Alvarenga M., Worcester M., Jackson A. (2020). Anxiety and Depression After a Cardiac Event: Prevalence and Predictors. Front. Psychol..

[56] Murphy B.M., Higgins R.O., Shand L., Page K., Holloway E., Le Grande M.R., Jackson A.C. (2017). Improving Health Professionals’ Self-Efficacy to Support Cardiac Patients’ Emotional Recovery: The ‘Cardiac Blues Project’. Eur. J. Cardiovasc. Nurs..

